# Influences of COVID-19 pandemic on hospital-at-home functions in Finland – a questionnaire survey

**DOI:** 10.1080/02813432.2022.2139475

**Published:** 2022-11-03

**Authors:** Reino Pöyhiä, Antti Ohvanainen, Susanna Rapo-Pylkkö, Leila Niemi-Murola

**Affiliations:** a Department of Anaesthesia and Intensive Care, University of Helsinki, Helsinki, Finland; b Palliative Medicine, Department of Clinical Medicine, University of Eastern Finland, Kuopio, Finland; c Palliative Center, The South Savo Social and Health Care Authority, Mikkeli, Finland; d Palliative Center, Joint Municipal Authority for North Karelia Social and Health Service, Siun sote, Joensuu, Finland; e Palliative Center, Espoo City Hospital, Espoo, Finland

**Keywords:** COVID19, pandemic, hospital-at-home, health service research, infections

## Abstract

**Objective:**

To investigate functions of Finnish hospital-at-home (HAH) during the first year of COVID19-pandemic 2020 compared with the previous year 2019.

**Design:**

Retrospective questionnaire survey.

**Setting:**

Finnish HAHs from Northern, Eastern, Southern, Western and Central parts of Finland participated in a questionnaire web-based questionnaire survey. The numbers of patients, activities and staff in 2019 and 2020, participation in the care of COVID19 patients, availability of protective clothing, attitudes of patients towards home care and development of new practices in the corona era were asked using both predefined and free questions.

**Subjects:**

questionnaire was sent to the nurses and physicians in charge of the HAHs (*N* = 13), 77% responded. The HAHs provided services to a total of 1,196,783 inhabitants in their municipalities.

**Results:**

There were no significant changes in the numbers of patients, staff or activities between the years 2019 and 2020. Although nurses did viral tests, COVID19 patients were cared only in 40% of HAHs. Protective clothing was well available. New instructions for infection management were created.

**Conclusions:**

The COVID-19 pandemic did not largely influence the functions of the examined Finnish HAHs in 2020. Most activities and patients’ characteristics remained unchanged from 2019. The role of HAHs should be further developed in Scandinavian countries, particularly during pandemics.

**Key Points**
- Hospital-at-home (HAH) is a cost-effective model to provide hospital-like services.
- Data about the role of HAHs during COVID19 pandemics is lacking in the Nordic countries.
- This study shows that, the large Finnish municipal HAHs have been not influenced by pandemics.

## Introduction

There has been a substantial decrease in the use of many health care services not related to SARS-CoV-2 infection during the pandemic all over the world. For instance, delays in cancer diagnostics [1], reduced lists of surgery [2,3], diminished emergency services [4–7] and worsened care of chronic diseases have been reported [8,9]. According to the national health care report, referrals to the special health care were reduced by 7.8% and emergency services by 16% in average in 2020 from the previous year also in Finland [10]. The obvious reasons for this development are the lockdown of society, lack of health care professionals and prioritising pandemic-related care when allocating health-care resources [11,12].

However, the pandemic has also stimulated health care professionals to create new services, one example of which is using a hospital-at-home (HAH) model for creating a home-based haematology unit for myeloma patients and the other one is establishment of an emergency department/a home care-facility in Spain [13,14]. HAHs could also serve as an expansion to the hospital, providing care only for COVID-19 patients [15]. Examples of similar activities can also be found in the USA [16].

**Figure 1.**
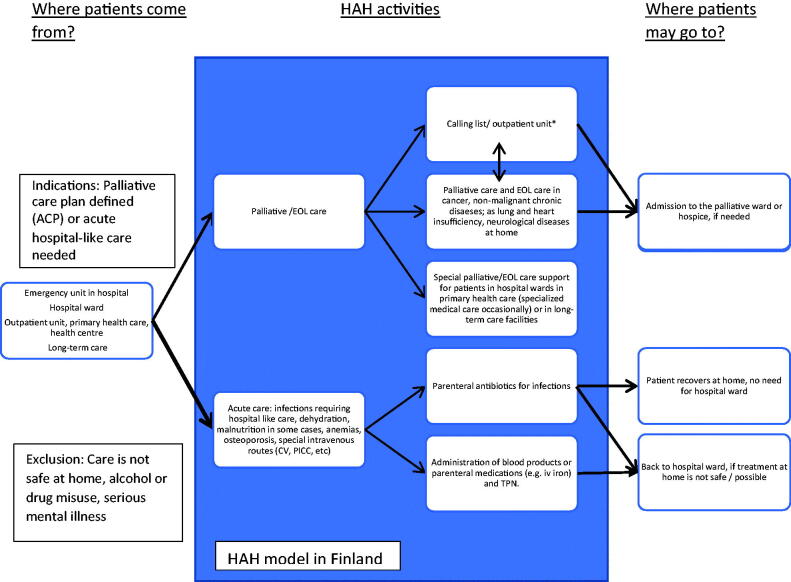
Basic structure of the HAH model in primary health care in Finland [17]. The arrowed lines present the flow of patients. Indications for eligibility and criteria for exclusion for HAH are given in the boxes. Main target is to produce care for adult patients otherwise requiring inpatient ward care and palliative care services. *Calling list includes patients, who are registered to a HAH but do not need regular visits to home. Some HAHs may also provide outpatient unit for policlinic visits. AC: advanced care planning; EOL: end-of-life.

We have recently described the national HAH network including 56 units as a part of Finnish public primary healthcare [17]. In comparison to home-based basic care, the services provided by the HAHs include more advanced, hospital-like care (Figure 1). The patients in Finnish HAHs may live either in their own homes or nursing homes. The service is available mostly to adults; there are only few HAHs for children. The recent study [17] identified two major services provided by the Finnish HAHs, which are the care of bacterial infections with parenteral antibiotics and end-of-life (EOL) care. However, nation-wide information about the HAHs’ functions during the pandemic are lacking in our country or other countries.

This study was carried out to investigate functions of Finnish HAHs during the first year of COVID-19 pandemic (2020) compared with the previous year 2019.

## Population and methods

According to the consultation of Ethical Review Board in the Humanities and Social and Behavioural Sciences of the University of Helsinki no formal assessment of ethical committee was needed. A web-questionnaire with predefined and open-ended questions on the practices of HAHs in 2019 and in 2020 were emailed to nurses and doctors in charge of 13 municipal HAHs in Finland. This convenience sample of HAHs was selected to represent various geographic regions of the country, providing services in the cities with more than 20,000 inhabitants but excluding the capital.

### Assessment of variables

The questionnaire (see Supplementary Appendix) included questions on the total numbers of patients, home visits, EOL care at home and nurses and physicians. Information was also gathered on any possible changes in the functions of HAH (yes/no), whether there were any COVID-19 patients (yes/no), nurses’ participating in corona-testing (yes/no), availability and use of protective clothing difficulties in getting staff (no difference from the previous year/significantly more difficult/cannot say), percentages (<30%, 30–50% or >50%) of staff at the most on sick-leave in 2020 and any observed differences in the attitudes of the patients on HAH activities in comparison to the previous year (yes/no). Furthermore, the responders were able to provide narrative information for reasons of changes in the services, on possible other changes and new ways to work in their HAH during the pandemic.

Since some HAHs reported nursing days of visits to one patient and some the actual number of home visits, these two variables were combined, and a new variable, activity units, was created. The median values with ranges given where appropriate. Pearson’s correlation test was used to study the association between the number of COVID-19 patients with the total number of patients and activities. Statistical analysis was executed with a free software copy of PSPP. To provide anonymity the HAHs were classified in three categories according to the size of their service area in the following way: population <50,000 (A), 50–100,000 (B) and >100,000 (C) in the municipalities of the HAHs.

The demographic and COVID-19 epidemic data concerning the municipalities of the HAHs were obtained from Statistics in Finland [18]. The cumulative numbers of COVID-19-infected patients in these municipalities at the end of 2020 were compared with the national ones. The data on the population of the municipalities where the HAHs included in the study are functioning were obtained from The Association of Finnish Local and Regional Authorities website [19].

## Results

Comprehensive responses were received from 10 HAHs from various parts of Finland, which represents 15% of all HAHs. A total of 1,196,783 people, corresponding to app. 22% of the Finnish population, lived in the catchment areas of these hospitals in the end of 2020. The cumulative number of COVID-19 cases in the municipalities of these HAHs was 9313 and that of the whole of Finland 36,653 by the end of 2020. The numbers of patients and activities of the HAHs are given in Table 1. The correlation between the number of COVID-19 cases and HAH activity numbers were insignificant (*r* = 0.26–0.28). Four HAHs had COVID-19 patients in active care and in eight HAHs the nurses performed COVID-19 tests on the patients.

**Table 1.** Activities of HAHs in Finland in 2019 and 2020. HAH = number of the responding HAH.

HAH	Part	Size	Patients 2019	Patients 2020	Activity 2019	Activity 2020	Act/pt 2019	Act/pt 2020	EOL 2019	EOL 2020	Deaths 2019	Deaths 2020
1	South	C	1500	1750	16,500	17,200	11	10	120	150	75	100
2	South	C	2049	1912	9893	9382	5	5	469	504	26	24
3	West	C	1014	1219	9872	12,382	10	10	–	–	–	–
4	West	B	708	708	10,497	9060	15	13	–	52	–	39
5	Middle	C	642	575	11,933	11,709	19	20	347	310	52	79
6	Middle	C	3167	2979	22,689	20,137	7	7	–	–	–	–
7	East	B	893	852	10,309	10,799	12	13	159	170	32	49
8	East	B	545	679	4450	4764^a^	8	7	46	57	13	15
9	North	B	640	570	11,933^a^	11,808	19	21	320	285	52	79
10	North	A	642	601	3524^a^	3714^a^	5	6	30	40	16	20
	Median		844	800	10,403	11,254	10	10	139	150	29	44

Part: location of the HAH in Finland. Size: Population of the catchment area: A = < 50,000; *B* = 50–100,000; C) > 100,000. *N* 2019/2020: numbers of patients in 2019/2020.

^a^Activity 2019/2020: number of patient visits or days of care.

Act/pt: activity per patient; EOL 2019/2020: numbers of patients in EOL care 2019/2020; Death 2019/2020: numbers of death in home-based EOL care

In the most HAHs the main activities were rather similar in both years (Tables 1 and 2). Five HAHs reported changes from the previous years: in one HAH the number of EOL care and infection patients had increased, in another HAH the number of infectious patients had decreased and in one HAH palliative care had increased in 2020 from the previous year. Increased demand for patients in nursing homes and old patients’ facilities was reported by two HAHs in 2020. Although the mean number of patients in EOL care declined, the average number of deaths at home increased in 2019 and 2020, respectively (Table 1).

**Table 2.** Median (range) numbers of nurses and physicians and HAHs with availability of staff and COVID-19-related actions.

Variable	2019	2020
NNurs	15 (5–29)	17 (7–29)
NPhys	1 (0.5–3)	1 (0.5–4)
Diff Nurs		1/4/5
Diff Phys		0/6/3
Staff on sick-leave		6/2/0
Corona sampling (N/Y)		2/8
Attitude changes (N/Y)		8/2

NNurs: number of nurses; NPhys: number of physicians; Diff Nurs: difficulties to get nurses: numbers of HAHs with no/no changes/more difficult than in 2019; Diff Phys: difficulties to get physicians: numbers of HAHs with no/no changes/more difficult than in 2019; Staff on sick-leave: numbers of hospitals with < 30/30–50/>50% on sick-leave at the most; corona sampling: numbers of HAHs where corona virus samples were taken by nurses on not; attitude: observed changes in patients’ attitudes on HAH.

N: no; Y: yes

There was no major lack of supply of standard protective clothing which included gloves, facial masks and protective gowns, which were the standard personal protective equipment in all units. New protocols on hygiene and other activities were created in three HAHs. Staff meetings were organised virtually. One HAH had introduced totally paperless way of functioning. Using the protective care had become as a standard procedure in all HAHs.

The staff in most HAHs had not observed significant changes in the attitudes of the patients towards home-based care. In one HAH the staff had observed patients expressing fear of catching the COVID-19 virus from the HAH nurses, and in another HAH patients were pleased to get health care services at their home.

## Discussion

Although Finland’s cumulative number of SARS-Co2 virus-infected individuals (36,653) at the end of 2020 was among the lowest ones in the world, a state of national emergency was declared from 16 March to 16 June 2020, including movement restrictions in the Uusimaa region between 28 March and 16 April 2020. Nevertheless, Finland experienced two peaks in the incidence of COVID-19 in 2020 [20–22]. Our study shows that despite of downshifting of many health care services, the HAH institution did not suffer from the pandemic. One of the reasons may be that HAHs had not the necessary competence at that time. The other reason may be that the central authorities were not fully aware of the existing HAH resources. Only in one HAH the staff had observed their patients expressing negative attitudes towards homecare.

We investigated the functions of HAHs in ten Finnish HAHs in areas the populations of which represent approximately 20% of the population of the country. The incidence of new COVID-19 cases varied largely in the areas of the HAHs included in the study, but all regions experienced all three phases of the epidemics (Table 3): baseline, acceleration and spreading phase during the first year of pandemics. Thus, we believe that our findings represent the situation in the entire country rather well. They are in accordance with the national gross-statistics, which show that the total number of patients in all reporting Finnish HAHs has increased from 6635 in 2019 to 10,975 in 2020 [23]. The national statistics included a bigger number of HAHs than we did, and also capital HAH, which we did not. Several reasons may explain these findings. First, Finnish individuals may have been afraid of being cared for in the brick-and-mortar hospitals; second, resources in the brick-and-mortar hospitals were allocated to meet the increased need of beds for COVID-19 patients, and thirdly, the Finnish authorities strongly suggested especially elderly people to limit their movement in crowded spaces outside their homes.

**Table 3.** Finnish classification of the severity of COVID-19 pandemics.

State of pandemic	Definition
Basic state	incidence is low local and regional chains of infections can be traced effectively significant spread of infection is not detected outside the chains new cases are sporadic or in the quarantines
Acceleration: epidemics are accelerating from the baseline	the regional incidence of infections is > 10–25 per 100,000 persons over a period of 14 d. local and regional transmission chains are largely traceable there is sufficient capacity to respond to the need for hospital care without special measures
Spreading phase: infections are spreading at the regional level or more broadly throughout the population, tracing is not possible	incidence of > 18–50 infections per 100,000 persons over 14 d. increase in daily incidence rate > 10%. < 50% sources of infection are traceable, the need for hospital care and intensive care is growing sharply

We were surprised to notice that care of COVID-19 patients was not largely included in the activities of the Finnish HAHs. In Finland, at least in the beginning of the epidemic, patients were rather easily transferred to hospitals. Only patients with mild symptoms were advised to stay at home under telephone surveillance from the primary health care unit. The possibilities of HAHs to provide more advanced COVID-19 care with remote controlling units have not been recognised in Finland. However, experiences both in Spain and the USA have shown that a HAH-model could be useful and cost-saving in the care of patients with mild to moderate pneumonia due to COVID-19 [14,16,24–26]. This approach requires both remote monitoring and clear criteria for transmission to hospital.

In Finland, the HAHs seem to have provided important care for patients with other infections than COVID-19 and at EOL care, to avoid hospitalisation. So far, only few teams have reported opening HAH services for cancer care [13,27]. Undoubtedly, HAH could be an important mechanism of healthcare service delivery for different types of care in order to avoid brick-and-mortar hospitalisation and thus saving those resources during pandemics. As an example, HAH could be used for postoperative rehabilitation and cancer treatment more effectively than nowadays [17,28,29]. Care at home definitely reduces personal contacts of patients to other individuals and thus risk of viral contamination. Naturally, the development of HAH care would need access to functional remote monitoring equipment [30,31].

The COVID-19 pandemic and its consequences have undoubtedly led to an increased rate of burnout and stress among healthcare-workers [32]. Burnout or psychological problems among the staff were not reported spontaneously in our study. Although the HAHs had struggled more than previously with employing staff, they did not really seem to suffer from any lack of staff. HAHs might offer a more attractive alternative for healthcare professionals who would not like to work in the brick-and-mortar hospitals particularly during the pandemic. In addition, HAH as a work environment may be experienced as less stressful than a brick-and-mortar hospital, working in which has been shown to increase staff’s anxiety during pandemic. Significantly increased stress levels have been reported also among patients during the corona era [12]. Yet in our study only one HAH had noticed patients being suspicious towards the HAH.

Although we were able to collect data from HAHs covering approximately 20% of the Finnish population, our sample is still relatively small. In addition, we did not include smaller HAHs in our study. Yet, our sample included roughly a quarter of all public HAHs. Another limitation is the qualitative nature of the information. In order to provide anonymity, we decided not to gather exact numbers of corona patients. Undoubtedly, there is room for more detailed studies about the role of HAHs in Finland during the COVID-19 and other pandemics in the future. Our study also shows the lack of similar data recording in HAHs, which would be important for both for quality assessment and systematic development of services, and facilitate prospective studies. The role of HAH remains unexplored also in other Scandinavian countries.

## Conclusions

The HAHs in Finland have remained largely unaffected by the COVID-19 pandemic [17]. The functions of the HAHs as well as the patient characteristics have remained rather unchanged in 2020 in comparison to the year before. Obviously, the resources of HAHs could be more actively utilised both in care for non-infected and infected patients. This would also necessitate systematic education of HAH staff on care during pandemics. Future studies are needed to assess the role of HAH during pandemics.

## Supplementary Material

Supplemental Material
