# Supplementary material for: Influences of COVID-19 pandemic on hospital-at-home functions in Finland – a questionnaire survey
Source: Scand J Prim Health Care. 2022 Nov 3;40(3):379–84. doi: 10.1080/02813432.2022.2139475 (PMC9848305; doi:10.1080/02813432.2022.2139475)
Supplement: Supplemental Material [file IPRI_A_2139475_SM2722.pdf]

Arvoisa osastonhoitaja/ylihoitaja

Olemme aikaisemmin osoittaneet tutkimuksessamme Suomen upea kotisairaalaverkoston rakenteen ja potilasaineiston sekä hallinnollisesti kovin kepeän rakenteen. Kotisairaalamme tekevät korvaamattoman arvokasta työtä hyvin aliresurssoituina.

Pyydämme Sinua nyt mukaan uuteen kyselytutkimukseen. Haluamme selvittää, onko koronavirus-epidemia vaikuttanut suomalaisten kotisairaaloiden työhön. Lähetimme kyselyn word-dokumenttina muutama viikko sitten. Nyt siihen on web-vastauslinkki:

Suurimpaan osaan kysymyksistä vastataan numerotiedoin tai valitsemalla sopivin vaihtoehto, mutta laadullista tietoa voit meille välittää myös vapaalla tekstillä. Arviomme mukaan lomakkeen täyttöön kuluu 15-30 min aikaa. Osallistuminen on vapaaehtoista. Halutessasi voit vielä vastattuasikin perua tietojen käytön. Tiedot analysoidaan ja julkaistaan siten, että kotisairaaloita ei vastauksista voi tunnistaa. Helsingin yliopisto eettinen toimikunta on arvioinut tutkimuksen. Lisätietoja saat dos Reino Pöyhiältä.

Olemme kiitollisia, jos voisit vastata oheisen linkin kysymyksiin mahdollisimman pian!

Halutessasi lähetämme julkaisun aikanaan SInulle!

Taustatiedot

1 a. Kuinka monta eri potilasta hoidettiin kotisairaalassasi vuonna 2019 (1.1. - 31.12.2019)?

1 b. Kuinka monta eri potilasta hoidettiin kotisairaalassasi vuonna 2020 (1.1. - 31.12.2020)?

2 a. Mikä oli kotikäyntien määrä (hoitajat ja lääkärit yhteensä) vuonna 2019?

2 b. Mikä oli kotikäyntien määrä (hoitajat ja lääkärit yhteensä) vuonna 2020?

3. Montako potilasta oli kotisairaalasi kotisaattohoidossa (yksityiskodit ja palveluasumisyksiköt) vuonna 2019?

Montako heistä kuoli kotona?

4. Montako potilasta oli kotisairaalasi saattohoidossa vuonna 2020?

Moniko heistä kuoli kotona?

Potilaat pandemian aikana

5. Muuttuiko potilasaineisto vuonna 2020 jollakin tavoin edelliseen vuoteen verrattuna? -/ei/kyllä

Jos vastasit kyllä, voitko kuvailla miten?

6. Hoidettiinko kotisairaalassasi COVID-19 potilaita vuonna 2020? -/ei/kyllä

7. Oliko kotisaattohoidossa COVID-19-potilaita vuonna 2020?

8. Muuttuiko potilaiden suhtautuminen kotisairaalaan edellisestä vuodesta? -/ei/kyllä

Jos muuttui, niin miten? Kerro vapaasti!

Henkilökunta

9. Mikä oli sairaanhoitajien kokonaismäärä vuonna 2019?

10 b. Mikä oli sairaanhoitajien kokonaismäärä vuonna 2020?

10 c. Mikä oli lääkärien kokonaismäärä vuonna 2019?

10 d. Mikä oli lääkärien kokonaismäärä vuonna 2020?

11. Oliko sairaanhoitajien sijaisten saaminen 2020 yhtä helppoa kuin 2019?

-/en osaa sanoa/ei eroa aikaisempaan vuoteen/paljon vaikeampaa kuin aikaisemmin koska hoitajista oli pula

12. Oliko lääkärien saaminen kotisairaalaan vuonna 2020 yhtä helppoa kuin 2019?

-/en osaa sanoa/ei eroa aikaisempaan vuoteen/paljon vaikeampaa kuin aikaisemmin koska lääkäreistä oli pula

13. Oliko kotisairaalan henkilökunta työstä pois COVID-19 takia (sairastuneita tai karanteenissa)

-/enimmillään < 30 %/30-50 %/> 50 %

14. Ottivatko kotisairaalan sairaanhoitajat korona-näytteitä?

-/ei/kyllä

Materiaalien saatavuus

13. Miten kotisairaalan henkilökunta suojautui kotikäynnillä?

☐ a. Kirurginen tai muu maski

☐ b. Käsineet

☐ c. Käsien desinfiointi

☐ d. Suojatakki/esiliina

☐ e. muu

14. Oliko kotisairaalassa riittävästi suojaimia vuonna 2020?

-/ ei koko vuonna/kyllä, alussa /kyllä, koko ajan

14 b. Jos on ollut hankaluuksia suojainten saannissa, koska vaikeudet helpottivat?

Katse tulevaisuuteen

15. Miten pandemia on vaikuttanut kotisairaalan työhön? Muuttuikó jokin? Kerro vapaasti!

16. Tuliko toimintaan vuonna 2020 jokin uusi toimintatapa tai malli, jonka arvelet jäävän pysyväksi kotisairaalassasi?

Yhteystiedot

Kotisairaalan nimi
